# Optical Coherence Tomography in the Differential Diagnosis of Patients with Multiple Sclerosis and Patients with MRI Nonspecific White Matter Lesions

**DOI:** 10.3390/s21217127

**Published:** 2021-10-27

**Authors:** Małgorzata Siger, Marta Owidzka, Mariola Świderek-Matysiak, Wojciech Omulecki, Mariusz Stasiołek

**Affiliations:** 1 Department of Neurology, Medical University of Lodz, 90-419 Lodz, Poland; mariola.swiderek-matysiak@umed.lodz.pl (M.Ś.-M.); mariusz.stasiolek@umed.lodz.pl (M.S.); 2 Department of Eye Disease, Medical University of Lodz, 90-419 Lodz, Poland; marta.owidzka@umed.lodz.pl (M.O.); wojciech.omulecki@umed.lodz.pl (W.O.)

**Keywords:** multiple sclerosis, nonspecific white matter lesions, differential diagnosis, optical coherence tomography

## Abstract

In the differential diagnosis of nonspecific white matter lesions (NSWMLs) detected on magnetic resonance imaging (MRI), multiple sclerosis (MS) should be taken into consideration. Optical coherence tomography (OCT) is a promising tool applied in the differential diagnostic process of MS. We tested whether OCT may be useful in distinguishing between MS and NSWMLs patients. In patients with MS (n = 41) and NSWMLs (n = 19), the following OCT parameters were measured: thickness of the peripapillary Retinal Nerve Fibre Layer (pRNFL) in superior, inferior, nasal, and temporal segments; thickness of the ganglion cell-inner plexiform layer (GCIPL); thickness of macular RNFL (mRNFL); and macular volume (MV). In MS patients, GCIPL was significantly lower than in NSWMLs patients (*p* = 0.024). Additionally, in MS patients, mRNFL was significantly lower than in NSWMLs patients (*p* = 0.030). The average segmental pRNFL and MV did not differ between MS and NSWMLs patients (*p* > 0.05). GCIPL and macular RNFL thinning significantly influenced the risk of MS (18.6% [95% CI 2.7%, 25.3%]; 27.4% [95% CI 4.5%, 62.3%]), and reduced GCIPL thickness appeared to be the best predictor of MS. We conclude that OCT may be helpful in the differential diagnosis of MS and NSWMLs patients in real-world settings.

## 1. Introduction

An early introduction of efficient disease-modifying therapy (DMT) in multiple sclerosis (MS) has been shown to be crucial for long-term clinical outcomes [1,2,3]. Accordingly, substantial effort has been put into the improvement of diagnostic tools and criteria to facilitate the diagnostic process [4,5]. Nevertheless, the diagnosis of MS remains complex and difficult in many cases, and high percentages of misdiagnoses have been described in both North American and European populations [6,7,8,9]. Although magnetic resonance imaging (MRI) represents the most important paraclinical tool in the diagnostic process of MS, its specificity is not satisfactory [5]. Focal white matter lesions similar to those observed in MS have been described in many other neurological disorders, such as neuromyelitis optica spectrum disorders (NMOSDs), Sjögren’s syndrome, systemic lupus erythematosus (SLE), Susac syndrome and vasculitis, as well as in patients with migraines, small vessel disease (SVD), and cardiovascular risk factors [10,11]. Moreover, the constantly increasing availability of MRI results in the frequent detection of incidental, nonspecific white matter lesions (NSWMLs) in patients with otherwise subtle or no clinical symptoms [10]. In radiological reports, NSWMLs are defined as demyelinating, inflammatory, ischaemic, post-ischaemic, or unidentified lesions. The unknown origin of NSWMLs may cause significant concerns with regard to their potential diagnostic meaning and may lead to the improper application of the McDonald criteria [6,7,8]. Most importantly, the misinterpretation of MRI findings is one of the main factors contributing to the misdiagnosis of MS [6,7,8,9]. Unfortunately, there are currently no specific tests or sets of criteria that would allow for fully efficient discrimination between MS lesions and NSWMLs in clinical practice.

Optical coherence tomography (OCT) is a non-invasive ocular imaging tool that enables the evaluation of distinct layers of retina [12]. In recent years, growing evidence has been gathered in support of the utility of OCT as a tool in the diagnostic process and clinical assessment of patients with various central nervous system (CNS) diseases, including different demyelinating conditions [12,13,14,15,16,17].

In our recently published study, we evaluated the applicability of OCT in the differentiation between MS and autoimmune connective tissue disease (CTD) patients with CNS involvement [17]. Although we did not find significant differences between MS and CTD patients, in our study, retinal parameters distinguished patients with inflammatory CNS conditions from healthy controls [17]. Based on these observations, we decided to focus on the potential role of OCT as a paraclinical tool helpful in the differential diagnostics of MS and patients with NSWMLs detected on MRI.

## 2. Experimental Section

### 2.1. Study Design

The study was conducted according to the guidelines of the Declaration of Helsinki (1964) and its later amendments and approved by the Local Ethics Committee of the Medical University of Lodz (approval number/360/17/KE, 21 November 2017, RNN/231/18KE, 12 June 2018). Informed consent was obtained from all the subjects involved in the study.

The participants were consecutively recruited from the Department of Neurology and Neurology Outpatient Clinic, Medical University of Lodz, Poland, between December 2017 and August 2018. The participants had to be 18–55 years old and clinically stable, i.e., without any exacerbation of neurological signs and symptoms at least 6 months before enrolment in the study.

The subjects were divided into two study groups.

Group 1 included patients diagnosed with MS according to the McDonald Criteria 2017 (MS group). Only patients with the relapsing–remitting course of the disease were recruited for the study. Demographic and medical data, including clinical presentation of MS, disease duration, relapses, current and prior disease-modifying treatment (DMT), and comorbidities, were collected in a medical database. The level of neurological disability was assessed with the Expanded Disability Status Scale (EDSS) [18].

Group 2 included patients with NSWMLs detected by cerebral MRI (NSWMLs group). Two independent, trained, and certified radiologists identified NSWMLs according to previously published criteria [19,20] as supratentorial white matter lesions located mainly in paraventricular and subcortical areas not reaching the CSF space, with spotty appearance, not compatible with demyelinating disease. The main indications for MRI examination in this group of patients were unspecific, subjective complaints, such as transient and global weakness (n = 7), short-lasting incidents of vertigo (n = 8), and temporary mood decline (n = 4). Neurological examination in all participants was normal.

Exclusion criteria encompassed treatment with immunomodulatory and immunosuppressive drugs (other than DMTs in MS group), diabetes mellitus, hypertension, migraine, history of stroke or transient ischaemic attack, infectious, metabolic, toxic and metastatic diseases, systemic lupus erythematosus (SLE), Sjögren’s syndrome, neurosarcoidosis, rheumatic arthritis, psoriasis, undifferentiated connective tissue disorders, vasculitis, and dementia.

Additionally, exclusion criteria included conditions that could affect retinal parameters or the quality of OCT measurement, such as age-related macular degeneration and pathological retinal findings on ophthalmologic examination, such as glaucoma, hypertensive or diabetic retinopathy, post-cataract extraction, central serious chorioretinopathy, high refractive error (±6.00 D spherical equivalent), optic disc drusen, and a history of optic neuritis (ON). ON was identified on the basis of clinical information from medical records according to the accepted recommendations [21], visual evoked potentials, and the results of ophthalmologic examination, including colour perception, pupillary light reflex, best-corrected visual acuity, intraocular pressure, slit lamp examination of anterior and posterior segments, and OCT assessment (asymmetry with cut-off values of ≥4 µm for ganglion cell-inner plexiform layer (GCIPL) and ≥5 for retinal nerve fiber thickness in the peripapillary area (pRNFL)), according to published guidelines [22].

### 2.2. Data Collection

#### 2.2.1. Magnetic Resonance Imaging

All participants underwent brain MRI exams performed on a 3.0 T scanner (Vida, Siemens, Munich, Germany) according to the guidelines of the Polish Neurological Society and Polish Medical Society of Radiology [23]. The MRI protocol included the following sequences: axial 3D T1-MPRAGE (TR = 2200 ms, TE = 246 ms, TI = 900 ms, slice thickness = 1.5, number of slices = 167, pixel size = 1 × 1 × 1 mm), fluid-attenuated inversion recovery (FLAIR) (TR = 2560 ms, TE = 135 ms, TI = 6700 ms, slice thickness = 3.0 mm, number of slices = 46, matrix = 256 × 256), PD/T_2_-weighted (TR = 2560 ms, TE1/TE2 = 90/30 ms, slice thickness = 3.0 mm, number of slices = 46, matrix = 256 × 256), double-inversion recovery (DIR) (TR = 2560 ms, TE = 60, T11/TI2 = 4500/5700 ms, slice thickness = 3.0 mm, number of slices = 46, matrix = 256 × 256), and 3D T_1_-MPARAGE after contrast administration (gadolinium 0.1 mmol/kg.m.c.). MRI data were assessed by experienced radiology specialists blinded to the identity of the study participants and OCT findings.

**Figure 1:**
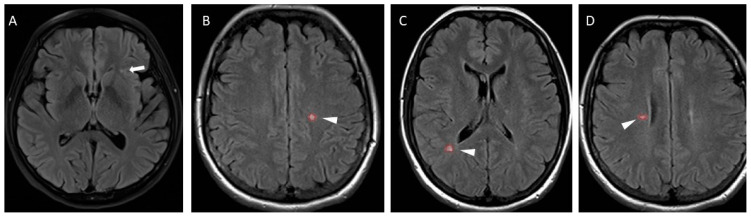
MRI images of multiple sclerosis (MS) and nonspecific white matter lesion (NSWMLs) patients. (**A**) Axial fluid-attenuated inversion recovery (FLAIR) images of NSWMLs patients showing a subtle white matter lesion located in deep white matter (white arrow). (**B**–**D**) Axial FLAIR images with hyperintense lesions in typical locations for MS (arrowhead).

Our NSWMLs patients were distinguished from radiologically isolated syndrome (RIS) patients following careful visual inspection. According to the exclusion criteria of RIS [24,25], all of our NSWMLs patients were classified as non-RIS patients. The quantification of the amount of NSWMLs was based on the Fazekas score [26] by two trained and certified radiologists who were blinded to the patients’ clinical information. All NSWMLs were classified as grade 1. Examples of MRI images in MS and NSWMLs patients are presented in Figure 1.

#### 2.2.2. Optical Coherence Tomography

Optical coherence tomography was performed using spectral-domain OCT (S-OCT) (Copernicus Plus device, software version 5.0, OPTOPOL Technology, Zawiercie, Poland, center wavelength: 840 nm, bandwidth: ±50 nm, resolution 5 μm). OCT was performed on the same day as neurological examination by one of the co-authors (M.O.), a blinded ophthalmologist with long-term experience in OCT examination (without pupil dilatation on both eyes in each patient). Each scan was assessed for proper fixation and quality. The retinal layers were also checked for errors in segmentation. To ensure proper quality of the image and reliability of the results, the following selection criteria were adopted:

- Quality index of the scan > 6—all scans included in the study;
- Quality index of the scan < 6—only scans with a clear and complete examination area included; and
- Quality index of the scan < 4—scan repeated or excluded from the study.

**Figure 2:**
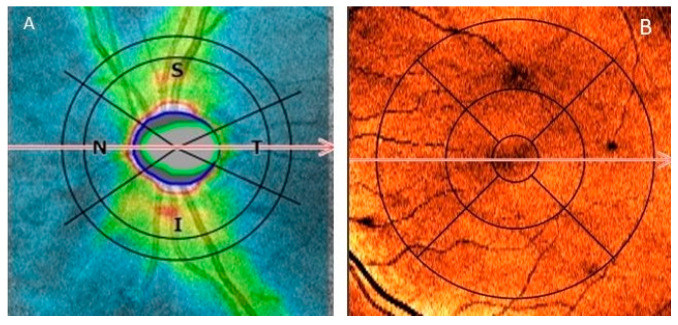
Representative optical coherence tomography (OCT) images of inspected regions (**A**). Position of the ring scan to measure peripappilary retinal nerve fiber layer thickness with superior (S), temporalis (T), inferior (I) and nasalis (N) quadrants. (**B**). Position of the macular subfields with inner, intermediate and outer rings to calculate average macular volume.

OCT imaging of the optic disc and the peripapillary area and macula was acquired with the 3D scanning protocol. The scan dimensions for the optic disc and peripapillary area were 5 × 5 mm, and those for the macula were 6 × 6 mm. To assess pRNFL thickness the middle of the ring scan (an internal diameter of 2.4 mm, width of 0.4 mm) was positioned automatically in the centre of the optic disc (Figure 2A). To measure average macular volume (MV), volumes of 3 subfields obtained using inner (diameter 1mm), intermediate (diameter 2.22 mm) and outer (diameter 3.45 mm) rings were summarized (Figure 2B). Copernicus Plus device, software version 5.0 automatically analysed the thickness of the retinal layers from the inner limiting membrane (ILM) to the retinal pigment epithelium (RPE). Detailed information concerning segmentation is provided in Supplementary Material and Figure S1.

Using available software, the following OCT parameters were calculated:

- Average thickness in pRNFL;
- pRNFL thickness in the superior, inferior, nasal, and temporal segments;
- Average GCIPL thickness (because of the low contrast between the ganglion cell layer and the inner plexiform layer, these two layers were combined to form the GCIPL [27,28]);
- Average macular RNFL thickness (mRNFL); and
- Average MV.

RNFL scans were performed using a pre-set protocol launched by the OCT user interface. The examined eye was fixed on an internal light, and a high-speed circle scan with a 3.40 mm diameter and 0.55 mm thickness centred on the optic nerve head was performed (automatic real-time AR 100). Only subjects with OCT measurements of both eyes were included in the study. Final values for all parameters were assessed as the mean from the values of both eyes. OCT results are referred to as the normative base for Caucasians. All examinations were checked for sufficient quality using the OSCAR-IB criteria [29] and APOSTEL recommendations [30].

### 2.3. Statistical Analysis

Analyses were carried out using statistical software R (version 3.5.2; a language and environment for statistical computing. R Foundation for Statistical Computing, Vienna, Austria). Data were assessed for normality using the Shapiro–Wilk test. Nominal variables are presented as *n* (%) and continuous variables are presented as the mean (±SD) or median (Q1; Q3), depending on the distribution of data. The primary analysis of interest included a comparison of MS and NSWML patients. For group comparisons, we used the chi-square test for nominal variables and the independent samples *t*-test or nonparametric Mann–Whitney U-test for continuous variables, as appropriate. Two-sided *p* < 0.05 was considered to indicate significant differences. Additionally, logistic regression was used to identify a combination of variables that differentiated the MS and NSWML groups. Only variables that were significantly different between groups were used as predictor variables in regression models. Age was included in the models as a covariate. Because logistic regression was used, model coefficients are in log odds form, so when the predictors increased by one unit, the outcome increased by log odds. These odds, in turn, were exponentiated into odds ratios (ORs), so when the predictors increased by one unit, the expected change in outcome was described in terms of odds. Model assessment was conducted with the χ^2^ test, Nagelkerke’s *R^2^* coefficient, and the Hosmer–Lemeshow goodness-of-fit (GOF) test.

## 3. Results

### 3.1. Patient Characteristics

This was a prospective cohort study of 60 patients, 41 with MS (male/female = 10/31) and 19 with NSWMLs (male/female = 5/14). There were no significant differences between groups as a function of age (*p* = 0.418) and sex distribution *(p* > 0.999). The clinical characteristics of MS and NSWML patients are presented in Table 1.

### 3.2. OCT Results

**Figure 3:**
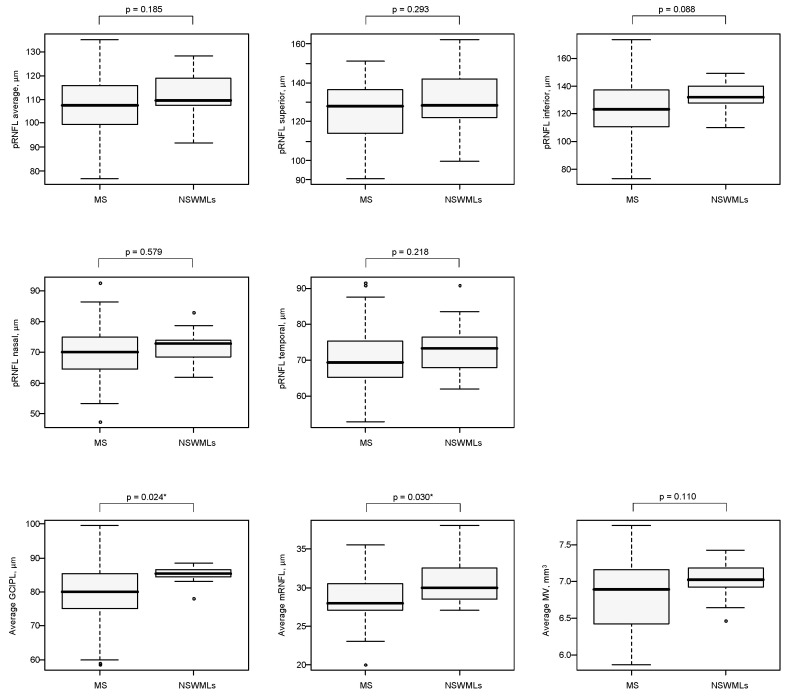
The boxplots of OCT parameters measured in MS and NSWMLs groups. Abbreviations: MS, multiple sclerosis; NSWMLs, nonspecific white matter lesions; pRNFL, peripapillary retinal nerve fiber layer; GCIPL, ganglion cell inner plexiform layer; mRNFL, macular retinal nerve fiber layer; MV, macular volume. p values were obtained from the Mann–Whitney U test for continuous variables and χ^2^ test or Fisher exact test for nominal variables. * indicate significant difference (*p* ≤ 0.05).

Analysis of OCT data in MS and NSWMLs patients revealed lower average GCIPL thickness in MS patients than in NSWMLs patients (*p* = 0.024). Moreover, in our MS patients, the average macular RNFL thickness was significantly lower than that in NSWMLs patients (*p* = 0.030). Analysis of the average and segmental pRNFL thickness and average macular volume did not show any significant differences between MS and NSWML patients (*p* > 0.05). A summary of the OCT data and statistics is provided in Table 2 and Figure 3. Additionally, representative OCT and MRI image of MS and NSWMLs patients was provided in Supplementary Material (Figure S2).

Figure 3 Results of OCT examination for each measured layers in MS and NSWMLs patients.

**Figure 4:**
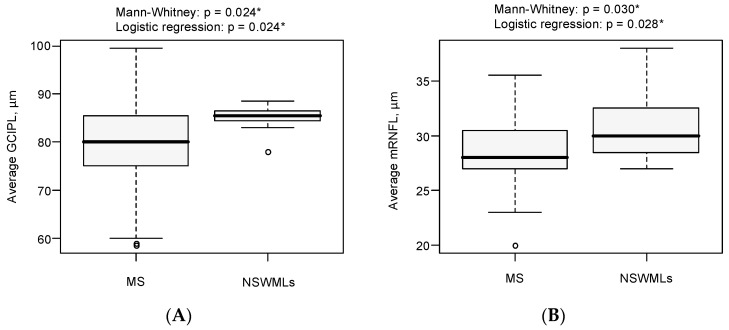
The boxplots of OCT parameters used to differentiate MS and NSWML groups. (**A**) GCIPL in the MS and NSWML groups, (**B**) macular RNFL in the MS and NSWMLs groups. *p*-values were obtained from the Mann–Whitney U-test and logistic regression model. The * indicates significant difference (*p* ≤ 0.05).

To identify a combination of variables differentiating the MS and NSWMLs groups, logistic regression was used. Parameters that were significantly different between MS and NSWML patients were used in the logistic regression model with MS as an outcome variable, including age as a covariate. The first step involved building a range of simple regression models with one predictor variable in each. According to the simple regression models, two parameters significantly influenced the risk of MS: GCIPL (*p* = 0.024) and mRNFL (*p* = 0.028). All parameters increased the risk of MS when they decreased in value by 1. GCIPL alone increased the risk of MS by 18.6% (95% CI 2.7%, 25.3%), and mRNFL alone increased the risk of MS by 27.4% (95% CI 4.5%, 62.3%). The next step involved a stepwise logistic regression including all the variables. According to this model, a decline in GCIPL is the best predictor of MS. Validation of the stepwise model with the χ^2^ test confirmed that the model was significant (*p* = 0.023). Model assessment with Nagelkerke’s *R^2^* coefficient showed that the model explained 25.8% of the data variation. The Hosmer–Lemeshow goodness-of-fit (GOF) test (*p* = 0.008) also confirmed a suitable fit of the model to the data. The results of the logistic regression model are presented in Table 3 and Figure 4.

Figure 4 Results of the logistic regression model.

## 4. Discussion

The highly heterogeneous and fluctuating symptomatology of MS and the lack of specific disease biomarkers make the diagnosis of MS challenging and complicated in many cases. In clinical practice, among various conditions that should be taken under consideration in the differential diagnosis of MS, an important and constantly growing group consists of patients with asymptomatic NSWMLs detected on MRI [6,7,31]. Although MRI is a widely accepted paraclinical tool in the diagnosis of MS, its main limitation is its low specificity [4,5]. In recent years, OCT has proven to be a promising diagnostic method and has been gradually incorporated in MS scientific and clinical research [12,32]. In this cross-sectional study, we aimed to investigate the applicability of OCT in the differential diagnosis of MS and NSWMLs patients. To our knowledge, this is the first study analysing the use of OCT in such clinical situations.

In our study, we demonstrated lower thickness of the GCIPL and mRNFL in MS compared with NSWMLs patients. Moreover, we found that thinning of the GCIPL and mRNFL increased the risk of MS diagnosis in our group of patients, while GCIPL thickness was the best OCT marker discriminating MS from NSWMLs patients.

pRNFL thickness represents the most intensively studied and widely used OCT parameter in MS [32,33,34,35]. The results of longitudinal studies showed the prognostic value of pRNFL thickness for the prediction of clinical progression (disability worsening and relapse) and its high sensitivity to inflammatory changes and damage caused by ON [33,36,37,38]. The unique structural composition of the pRNFL (the innermost retinal layer, composed of unmyelinated axons) makes the assessment of this structure an attractive marker of brain axonal degeneration [39,40]. This assumption is already supported by the results of studies evaluating the correlation between pRNFL thickness and brain and spinal cord atrophy [41,42,43,44]. One retinal structure that is less prone to the influence of changes associated with acute or remote (≥6 months) ON is GCIPL [45,46,47]. On the other hand, in the acute stage of ON, GCIPL thinning progresses faster than pRNFL thinning [48]. The available data also suggest that GCIPL thinning is more specific for MS [32]. A reduction in GCILP thickness in MS patients has been described in many studies [32,48]. The results indicate that GCIPL atrophy may act as a surrogate for an accurate estimate of neurodegeneration, neuroprotection, and/or remyelination in clinical practice and clinical trials [32,35,39]. It is also commonly accepted that GCIPL seems to be a promising marker of disease progression [49]. In a recently published study, Bstech et al. found that baseline macular GCPIL thickness ≤ 77 µm was associated with an increased risk of disability progression, and annual thinning of macular GCIPL cut-off ≥ 1 µm identified clinically progressing patients [49]. Assessment of MV is another standard parameter in OCT examination [32,39]. Recently published results indicate that MV loss may represent brain grey matter atrophy arising from retrograde degeneration from lesions in the optic nerves, chiasm, or tracts, which places the measure of the MV among possible markers of brain neuronal damage [39,50].

Although intensively studied, the application of OCT in the differential diagnosis of MS is not well established. The majority of published studies evaluate the role of pRNFL and GCIPL thickness measurements in the differential diagnosis of MS and other inflammatory CNS conditions, e.g., NMOSD, MOG antibody-associated disease, SLE, neurosarcoidosis, and Behcet’s disease [13,14,15,16,17,51]. In our recently published work, we assessed whether OCT measurements can provide a useful biomarker for distinguishing MS patients from patients with CNS involvement in the course of CTD [17]. Although there were no significant differences between MS and CTD patients, our analysis revealed clear differences in multiple OCT parameters between healthy controls and CTD patients.

To our knowledge, only few studies have analysed the use of OCT to evaluate retinal pathology in NSWMLs patients. Kim et al. [52] assessed the pRNFL on fundus photographs in patients with cerebral small vessel disease (SVD) with NSWMLs. They detected pRNFL damage in 5.4% of SVD patients. The risk of pRNFL damage was associated with the occurrence of MRI white matter lesions, hypertension, older age, and male sex. In another study, the authors investigated the thickness of retinal layers in patients with NSWMLs and healthy controls [53]. Thicknesses of the mRNFL and the GCIPL were significantly reduced in NSWMLs patients compared to healthy controls. Factors increasing the risk of macular RNFL thinning were older age, higher body mass index, and more advanced brain damage, as measured by the Fazekas score. A reduction in GCIPL thickness was associated with older age, a higher Fazekas score, and a history of smoking. The results from this study showed that degeneration of the retina measured by mRNFL and GCIPL thickness was associated with NSWMLs and deteriorated with the number of the lesions. Another recently published study, performed with OCT angiography, demonstrated correlations between pRNFL thickness and macular microvascular damage and the number of white matter lesions assessed on the Fazekas scale [54]. Although OCT is widely used in MS research and, as indicated above, few studies have addressed OCT in patients with NSWMLs, to our knowledge, there are no data concerning OCT application in the differential diagnosis of these two clinical conditions. In our study, we found that GCIPL thickness was significantly lower in MS patients than in NSWMLs patients. Although Knier et al. [20] described lower GCIPL thickness in RIS patients than in subjects with NSWMLs, in more recent literature, there are no reports describing a direct comparison of GCIPL between MS and NSWMLs patients.

One explanation of our finding is based on the well-recognised phenomenon of trans-synaptic degeneration [55,56,57,58,59]. Lesions in the optic radiation and visual cortex may prompt retrograde axonal degeneration and changes in deeper layers of the retina. An accurate analysis of lesion locations on MRI images of our population was not within the scope of this study, but we included MS patients with MRI images characteristic of MS (dissemination in space) with possible lesion locations in optic radiation and visual cortex. Another interpretation of the differences in GCIPL thickness between MS and NSWMLs patients is based on the commonly accepted theory that GCIPL thickness may provide an estimate of axonal neurodegeneration in MS patients [60,61,62], and we can assume that this pathomechanism has no application in NSWMLs patients.

We also found lower mRNFL thickness in MS patients than in NSWMLs patients. The previously mentioned study [20] reported a lower mRNFL volume in clinically isolated syndrome (CIS) and RIS patients than in NSWMLs patients. Lower mRNFL volume in RIS patients was associated with subsequent MRI activity and in CIS patients with conversion to clinically definitive MS. Interestingly, the authors did not observe any differences in OCT results between NSWMLs patients and healthy controls [20].

Surprisingly, we did not observe any differences in segmental and average thickness of the pRNFL between MS and NSWMLs patients. Thinning of the pRNFL has been observed in MS-ON and also in MS-NON eyes [39,60,63,64]. However, some results did not confirm these observations [39,64,65,66,67]. Data comparing pRNFL thickness in MS and NSWMLs patients are scarce. In the study by Knier et al., the authors did not find any differences in the pRNFL among RIS, CIS, and NSWMLs patients and healthy controls [20]. Accordingly, it was postulated that other OCT parameters, such as GCIPL or mRNFL thickness, may have greater value than pRNFL in the assessment of neuroaxonal damage in MS [39,60,66,67]. Anatomically, the pRNFL, macular RNFL, and GCIPL constitute one unit of the visual pathway. Any damage to this pathway gives rise to retrograde trans-synaptic degeneration, which causes atrophy of the inner retinal layers, mainly the GCIPL [39,60,66]. Moreover, growing evidence indicates that neuroaxonal damage is detectable faster as GCIPL thickness decreases with than changes of pRNFL [39,48,60,66]. The observed dissociation between changes in pRNFL thickness and other OCT parameters in MS patients may at least partially explain the results of our study. Support for this concept also comes from a study performed by Lotfy et al. [64], who found lower macular RNFL thickness in MS patients without ON compared with healthy controls, and no differences in pRNFL thickness between the two groups. Based on published results that show a better correlation of visual function and disability in MS patients and macular ganglion cell layer thickness than pRNFL [68], we can assume that in our MS patients, axonal degeneration may be more pronounced than in NSWMLs patients, and that the measurement of GCIPL and mRNFL thickness is more reliable than pRNFL in the differential diagnosis of MS. 

Surprisingly, we noticed that in our MS patients, MV was not different from that in NSWMLs patients. The evaluation of MV is a standard parameter in OCT examination [39,60]. In many studies, MV was lower in both MS-ON and MS-NON eyes than in healthy control eyes [17,39,60]. To the best of our knowledge, there is only one study comparing MV between NSWMLs patients and other groups. In the study performed by Knier et al., MV was comparable among RIS, CIS, and NSWMLs patients and healthy controls [20]. Additionally, in our recently published study [17], we did not detect a difference in MV between MS and CTD patients with CNS involvement and between the CTD and control groups. With respect to our actual findings, we postulate that assessment of MV is not specific enough to differentiate MS from NSWMLs patients.

The final step of our study was to identify an OCT parameter or combination of parameters that best discriminate MS from NSWMLs patients. We found that GCIPL thickness had the strongest value in the differentiation of MS and NSWMLs patients. The predictive value of GCIPL was investigated in different studies [60,66,67,68]. In the majority of studies, GCIPL was described as a suitable predictive marker of visual function after ON and neurological deterioration, as measured by the EDSS [66,67,68]. Additionally, in a recently published study [69], the inter-eye difference in GCIPL thickness differentiated MS patients in a large community cohort not only from healthy subjects but also from patients with other diseases. However, there is no information concerning patients with NSWMLs.

The main limitation of this study is the cross-sectional design and small sample size. We are also aware that we did not assess other retinal sublayers due to technical limitations of the software installed on our OCT device (reliably distinguishing between the GCL and the internal plexiform layer). Finally, we did not include a healthy control group in our analysis; however, OCT was performed by an experienced ophthalmologist using S-OCT, and the results were automatically compared with a normative database.

## 5. Conclusions

Despite the indicated limitations, we would like to stress that this is the first study to apply OCT in the differential diagnosis of MS and NSWMLs patients in a real-world setting. We conclude that OCT may be helpful as an easily accessible diagnostic tool in the differential diagnosis of MS, which may have implications for future therapeutic decisions.

## Figures and Tables

**Table 1:** Clinical characteristic of MS and NSWMLs patients.

.	MS	NSWMLs	*p-*Value
Subjects, *n*	41	19	
M/F	10/31	5/14	>0.999
Number of eyes	82	38	
Age, years, mean (±SD)	41.9 (±13.3)	43.8 (±12.0)	0.418
Disease duration, years, median (Q1; Q3)	3.0 (1.0; 7.0)	-	-
EDSS score, median (Q1; Q3)	1.0 (0.0; 3.0)	-	-

Groups were compared with the *t*-test for age and the chi-square test for sex. Abbreviations: MS, multiple sclerosis; NSWMLs, patients with nonspecific white matter lesions; M/F, male/female ratio; EDSS, Expanded Disability Status Scale; SD, standard deviation.

**Table 2:** Differences in OCT parameters of MS and NSWMLs patients.

Characteristic	MS (*n* = 41)	NSWMLs (*n* = 19)	*p-*Value
pRNFL average, µm	107.5 (99.5; 116.0)	109.5 (107.5; 119.0)	0.185
pRNFL superior, µm	128.0 (114.0; 136.5)	128.5 (122.0; 142.0)	0.293
pRNFL inferior, µm	123.0 (110.5; 137.0)	131.5 (127.5; 140.0)	0.088
pRNFL nasal, µm	70.0 (64.5; 75.0)	73.0 (68.5; 74.0)	0.579
pRNFL temporal, µm	69.5 (65.5; 75.5)	73.5 (68.0; 76.5)	0.218
Average GCIPL, µm	80.0 (75.0; 85.5)	85.5 (84.5; 86.5)	0.024 *
Average mRNFL, µm	28.0 (27.0; 30.5)	30.0 (28.5; 32.5)	0.030 *
Average MV, mm^3^	6.90 (6.43; 7.17)	7.03 (6.93; 7.19)	0.110

Data are presented as the median (Q1; Q3). * *p* < 0.05. Groups were compared with the Mann–Whitney U-test for continuous variables and the χ^2^ test or Fisher’s exact test for nominal variables. Abbreviations: MS, multiple sclerosis; NSWMLs, nonspecific white matter lesions; pRNFL, peripapillary retinal nerve fiber layer; GCLIPL, ganglion cell inner plexiform layer; mRNFL, macular retinal nerve fiber layer; MV, macular volume.

**Table 3:** Logistic regression model for MS and NSWMLs patients.

Characteristic	Simple Regression			Stepwise Regression		
	Coeff. (SE)	*p*	OR (95% CI)	Coeff. (SE)	*p*	OR (95% CI)
Average GCIPL, µm	−0.11 (0.05)	0.024	0.894 (0.798; 0.974)	−0.11 (0.05)	0.024	0.894 (0.798; 0.974)
Average mRNFL, µm	−0.24 (0.11)	0.028	0.785 (0.616; 0.957)	-	-	-
Constant				11.68 (4.73)	0.014	

Abbreviations: Coeff., beta coefficient of logistic regression; SE, standard error for coefficient; CI, confidence interval; OR, odds ratio, GCIPL, ganglion cell inner plexiform layer; mRNFL, macular retinal nerve fiber layer.

## Data Availability

The data presented in this study are available on request from the corresponding author. The data are not publicly available due to privacy issues.

## Supplementary Materials

Detailed information concerning segmentation, representative OCT and MRI image of MS and NSWMLs patients, Certificate of English language editing. The following are available online at https://www.mdpi.com/article/10.3390/s21217127/s1, Figure S1: Example OCT macular scan and segmentation by available Copernicus Plus software, Figure S2: Representative OCT and MRI images of patients with MS and NSWMLs.
