# Supplementary material for: Optical Coherence Tomography in the Differential Diagnosis of Patients with Multiple Sclerosis and Patients with MRI Nonspecific White Matter Lesions
Source: Sensors (Basel). 2021 Oct 27;21(21):7127. doi: 10.3390/s21217127 (PMC8588219; doi:10.3390/s21217127)
Supplement: Supplementary file 1 [file sensors-21-07127-s001.zip › sensors-1398135-supplementary.PDF]

2. Experimental section

2.2. Data collection

2.2.2. Optical Coherence Tomography

The automatic segmentation algorithm used in our study allowed to recognize following boundaries of retina layers (Figure S1)

- the inner limiting membrane (ILM) - red line (the innermost layer of the retina)

-the outer boundary of Retinal Nerve Fiber Layer (RNFL)- yellow line. The thickness of the RNFL is measured between the ILM and the outer boundary of RNFL.

-the outer boundary of the inner plexiform layer (IPL) -orange line. This part consists of ganglion cells (GCL) and IPL. Ganglion cells inner plexiform layer (GCIPL) thickness is measured between the outer boundary of RNFL and the outer boundary of IPL

-the outer boundary of inner nuclear layer (INL)- neon green line. This line separates the INL and the outer plexiform layer (OPL). The thickness of INL is measures between the outer boundary of IPL and outer boundary of INL.

-the outer boundary of OPL (violet line)- separates OPL and the photoreceptor cell bodies. The thickness of OPL is measured between the outer boundary of INL and the outer boundary of OPL

- photoreceptor inner segment/outer segment (IS/OS line)- green line. This line is located at the junction of the photoreceptor inner and outer segment. The thickness of outer nuclear layer is measured between the outer boundary of OPL and IS/OS line

-the retinal pigment epithelium (RPE). The inner boundary of RPE is marked as sky-blue line, outer boundary of RPE is marked as dark blue line. RPE is the outermost layer of retina, which is distinguished is segmentation.

Only 3D scanning protocol enables retinal thickness measurement. Software offers two algorithms of an indirect measurement of ganglion cell layer: RNFL+GCL+IPL and GCL+IPL and separately the RNFL thickness measurement. All mentioned methods were used in the study.

Figure S1. Example OCT macular scan and segmentation by available Copernicus Plus software

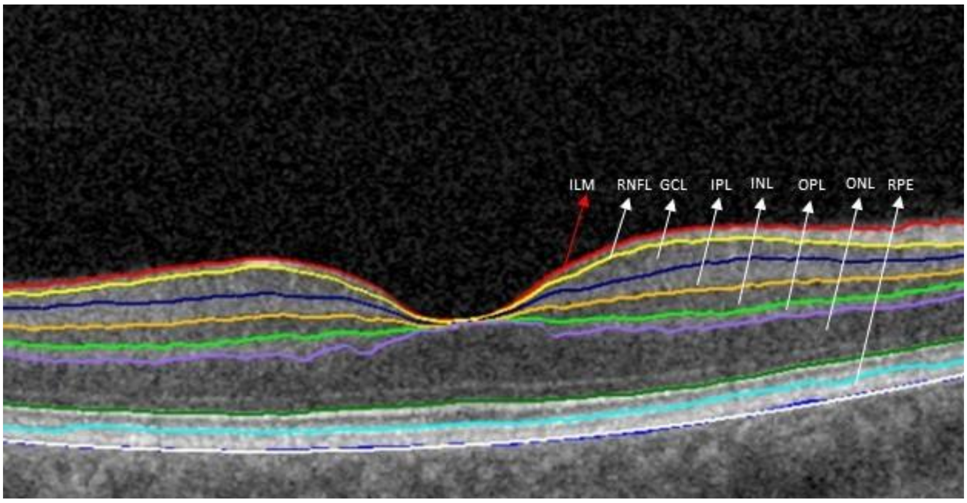

The red line is the inner limiting membrane (ILM). The retinal nerve fiber layer (RNFL) is between the red and yellow boundary line. The ganglion cell layer (GCL) is between the yellow and navy-blue boundary line. The inner plexiform layer (IPL) is between the navy blue and orange boundary line.
The inner nuclear layer (INL) is between the orange and neon green boundary line. The outer plexiform layer (OPL) is between the neon green and violet boundary line. The outer nuclear layer (ONL) is

between the violet and green boundary line. The retinal pigment epithelium (RPE) is between the sky-blue and dark blue line. The white line is parabolic fit for end of RPE layer.

3. Results

3.2. OCT Results

Figure S2. Representative OCT and MRI images of patients with MS and NSWMLs.

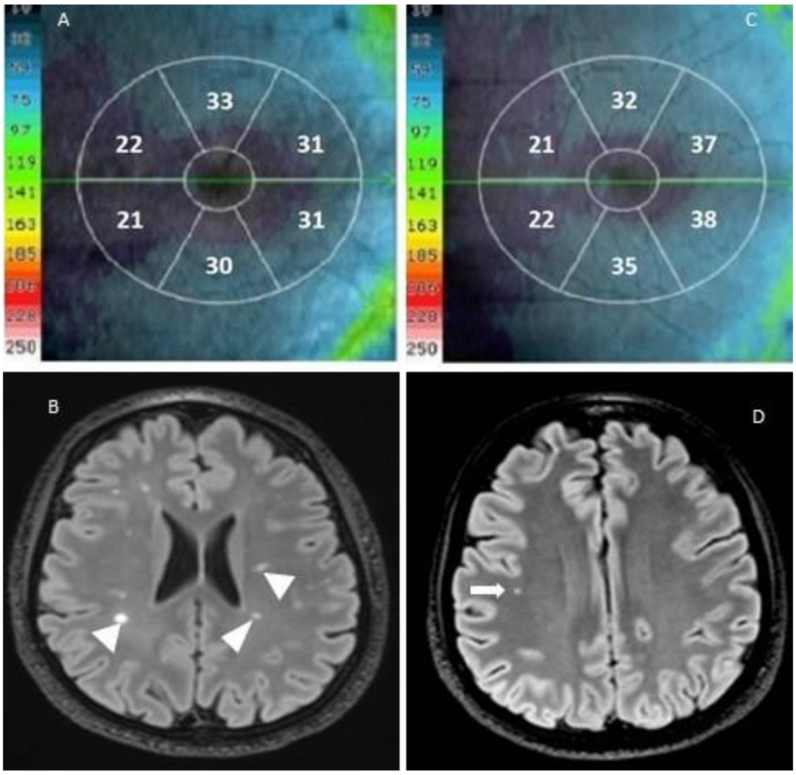

Figure S2. Representative optical coherence tomography (OCT) and MRI images of multiple sclerosis (MS) and nonspecific white matter lesion (NSWMLs) patients. (**A,C)** The colour-code macular retinal nerve fiber layer (mRNFL) thickness map (µm). The higher the value, the thicker the layer. (**A)** mRNFL thickness map of a patient with MS. (**C**) mRNFL thickness map of a patient with NSWMLs. (**B**) The corresponding axial fluid-attenuated inversion recovery (FLAIR) images with hyperintense lesions in typical locations for MS (*arrowheads*). **(D**) Axial FLAIR images of NSWMLs patient showing subtle white matter lesion located in deep white matter (*white arrow*).
